# A novel “twist-and-drag” loop clip technique for closure of mucosal defects after colorectal endoscopic submucosal dissection

**DOI:** 10.1055/a-2842-0664

**Published:** 2026-04-20

**Authors:** Tomoki Horikawa, Toshihiko Tomita, Shinichiro Shinzaki

**Affiliations:** 1 674409 Department of Gastroenterology, Hyogo Medical University School of Medicine, Nishinomiya, Japan

**Fig. 1:**
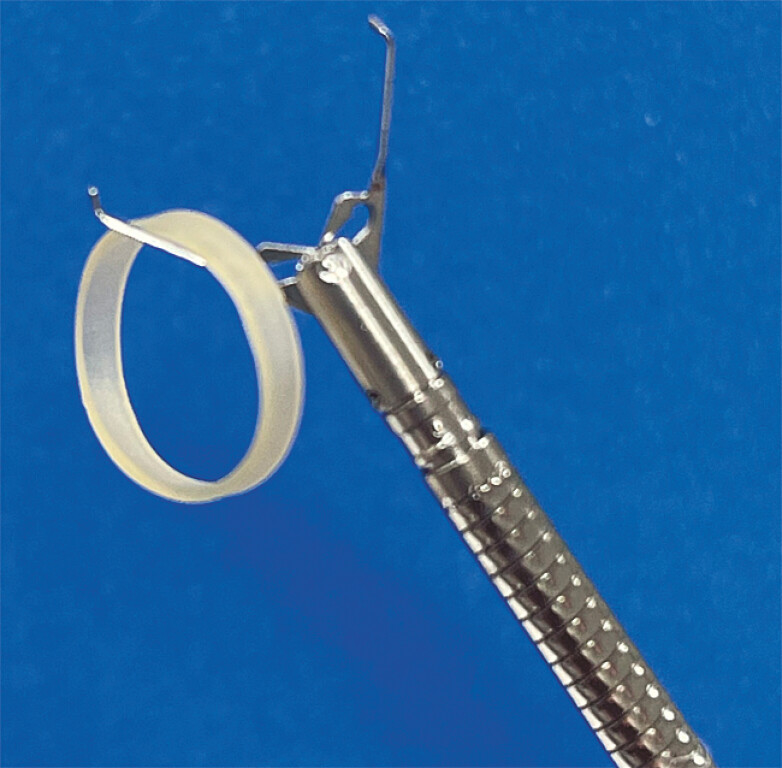
A clip with an 8-mm elastic band designed for orthodontic use.

Prophylactic closure of mucosal defects after colorectal endoscopic submucosal dissection (ESD) is expected to reduce delayed bleeding and perforation 1 2 . Therefore, various closure methods have been developed 3 4 5 , but these procedures still present challenges in terms of technical difficulty and cost. Herein, we invented a new closure method for large defects after ESD using the reopenable clip and an elastic band designed for orthodontics ( Fig. 1 ), which we have called “twist-and-drag loop clip closure” (TDL).

**Fig. 2:**
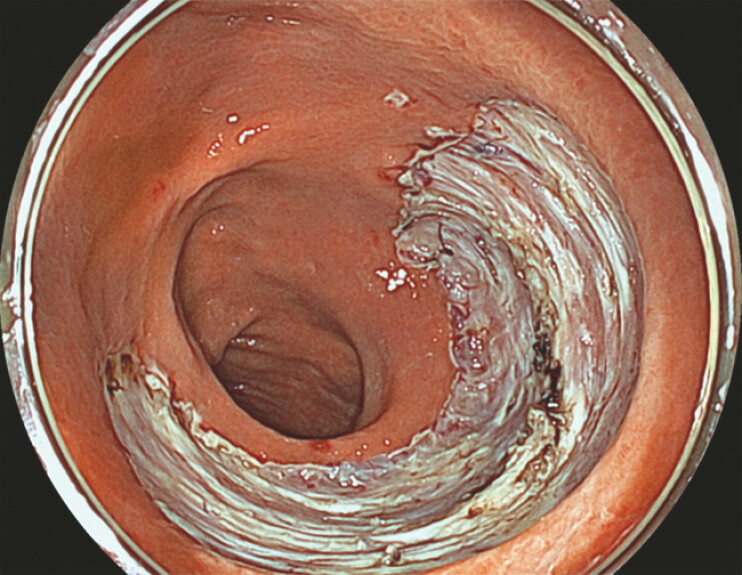
A 70-mm mucosal defect persisted after rectosigmoid endoscopic submucosal dissection.

**Fig. 3:**
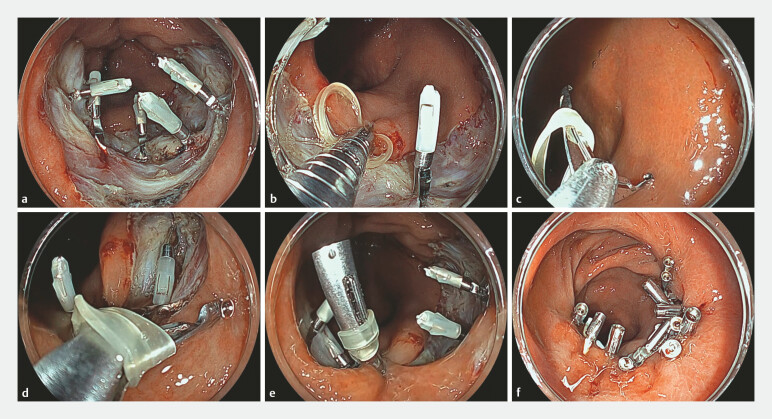
Twist-and-drag loop clip closure using double-layered suturing for large mucosal defects. **a** Conventional clips were applied directly to the muscular layer. **b** The clip with an elastic band was placed at the oral edge of the defect. **c** The elastic band was hooked using a reopenable clip, and the clip was twisted to wrap the elastic band around it. **d** The clip was dragged to the anal edge of the defect. **e** The clip was deployed after grasping both sides of the defect edge. **f** Complete closure of the mucosal defect was achieved by applying additional clips.

ESD was performed on a 65-mm intramucosal adenocarcinoma of the rectosigmoid colon, leaving a 70-mm mucosal defect ( Fig. 2 ). Endoscopic closure of the lesion was performed using the TDL method with double-layered suturing ( Video 1 ). First, to minimize submucosal dead space, several conventional clips were placed by grasping it directly in the muscular layer ( Fig. 3 **a** ). Next, the same clip was placed at the oral margin of the defect with an 8-mm elastic band (Tomy Inc., Tokyo, Japan; Fig. 3 **b** ). The elastic band was hooked using a reopenable clip (SureClip 16mm or SureClip eco 11mm; MC Medical, Tokyo, Japan), and the clip was twisted to wrap the elastic band around it ( Fig. 3 **c** ). The clip was dragged toward the anal margin of the defect, and both sides of the mucosa were grasped and closed ( Fig. 3 **d** and **e** ). This procedure was repeated several times, finally closing it completely with additional clips ( Fig. 3 **f** ).

Closure of large mucosal defects using the twist-and-drag loop clip closure method with double-layered suturing after rectosigmoid endoscopic submucosal dissection.Video 1

Conceptually similar to our approach is the hold-and-drag technique with an anchor-pronged clip 4 5 . Although the anchor-pronged clip is of high-cost, the elastic bands are inexpensive, and multiple procedures can be performed as needed. Furthermore, by wrapping the elastic bands around the clip, we can securely hold the defect edge and tightly sutured. The TDL method is a low-cost, simple, and effective technique for complete closure of large mucosal defects after colorectal ESD.

Endoscopy_UCTN_Code_TTT_1AQ_2AK
